# Endometrial Preparation for Frozen–Thawed Embryo Transfer With or Without Pretreatment With GnRH Agonist: A Randomized Controlled Trial at Two Centers

**DOI:** 10.3389/fendo.2021.722253

**Published:** 2021-10-18

**Authors:** Jian Xu, Shu-Zhen Li, Min-Na Yin, Pei-Ling Liang, Ping Li, Ling Sun

**Affiliations:** ^1^ Center of Reproductive Medicine, Guangzhou Women and Children’s Medical Center, Guangzhou Medical University, Guangzhou, China; ^2^ Reproductive Medicine center, Jiangmen Central Hospital, Affiliated Hospital of Sun Yat-Sen University, Jiangmen, China

**Keywords:** frozen embryo transfer, endometrial preparation, hormone replacement treatment, GnRH agonist, reproductive outcomes

## Abstract

**Objective:**

This prospective randomized controlled trial compared the reproductive outcomes of frozen embryo transfer (FET) with hormone replacement treatment (HRT) with or without gonadotropin-releasing hormone agonist (GnRHa) pretreatment.

**Methods:**

A total of 133 patients scheduled for HRT-FET mainly because of tubal and/or male factors who received two high-quality cleavage-stage embryos were enrolled at two participating centers. The GnRHa group (*n* = 65) received GnRHa pretreatment, while the control group (*n* = 68) did not. Analysis was based on the intention-to-treat (ITT) principle.

**Results:**

Among the 133 participants, 130 (97.7%) underwent embryo transfer and 127 (95.5%) completed the protocol. The clinical pregnancy rate according to ITT did not differ between the GnRHa and control groups [39/65 (60.0%) *vs*. 41/68 (60.3%), *p* = 0.887]. The implantation rate (47.6% *vs*. 45.3%, *p* = 0.713), early pregnancy loss rate (5.1% *vs*. 19.5%, *p* = 0.09), and live birth rate (49.2% *vs*. 50.0%, *p* = 0.920) were also comparable between groups.

**Conclusion:**

Pretreatment with GnRHa does not improve the reproductive outcomes for women receiving HRT-FET.

**Clinical Trial Registration:**

The study was registered with the Chinese Clinical Trial Registry (ChiCTR-IOR-17014170; http://www.chictr.org.cn).

## Introduction

Since the first successful case was reported in 1983, frozen–thawed embryo transfer (FET) has been used not only as a complement to stimulated *in vitro* fertilization (IVF) cycles but also is sometimes a routine procedure in IVF treatment (1). The advantages of FET cycles include higher cumulative pregnancy rates and lower IVF-associated complications such as ovarian hyperstimulation syndrome (2). Moreover, FET is associated with reduced risks of low or very low birth weight, small for gestational age infants, placenta previa, and placental abruption (3).

FET has been successfully performed in a natural cycle or after artificial preparation of the endometrium with consecutive estrogen and progesterone, which is known as hormone replacement treatment (HRT). Embryo transfer (ET) in a natural cycle has limited application in clinical practice as it requires more frequent visits to the hospital, is less flexible, and has a high risk of cycle cancellation (4).

In the artificial protocol, the cycle cancellation rate is drastically reduced, and physicians or patients can select the date of ET (4). HRT cycles can be performed with or without pituitary gland suppression induced by gonadotropin-releasing hormone agonist (GnRHa).

Administration of a GnRHa before HRT prevents spontaneous ovulation and cycle cancellation (5). Additionally, GnRHa was reported to enhance the expression of endometrial αvβ3 integrin, increase the number of pinopodes, and ultimately improve endometrial receptivity (6–11).

Several randomized controlled trials (RCTs) have investigated the potential benefits of GnRHa in HRT cycles, but there have been no definitive conclusions. Only one RCT reported higher pregnancy and live birth rates in women receiving GnRHa pretreatment (12); others have failed to demonstrate any benefits associated with GnRHa (13–19). It should be noted that, in these RCTs, the inclusion and exclusion criteria were not clearly defined and the protocols used for endometrial preparation differed in terms of drug type, route of administration, and dosage. Most importantly, embryo quality and number, which are critical for a successful pregnancy, were not among the inclusion criteria in these studies.

HRT-FET with GnRHa pretreatment was shown to be suitable for specific patient subgroups such as women with endometriosis or adenomyosis, as it is thought to transiently suppress the hypothalamic–pituitary–gonadal axis and exert a hypo-estrogenic effect (20, 21). A recent RCT of patients with polycystic ovarian syndrome (PCOS) receiving HRT showed that GnRHa pretreatment did not improve the pregnancy outcomes, but markedly increased the treatment costs (22). Most patients undergo IVF treatment because of inflammation or obstruction of the fallopian tubes, male oligospermia, asthenospermia, or dysspermia. However, it remains unclear whether GnRHa pretreatment is necessary with HRT-FET in these patients. To address this question, we conducted a RCT to compare the pregnancy outcomes of HRT-FET with or without GnRHa pretreatment.

## Materials and Methods

### Study Design and Participants

This RCT was conducted at the Department of Reproductive Medicine Center of Guangzhou Women and Children’s Medical Center and Jiangmen Central Hospital. Patients were enrolled from January 2018 to August 2020. The study was approved by the Independent Ethics Committee of Guangzhou Women and Children’s Hospital (#2018-02) and was registered with the Chinese Clinical Trial Registry (ChiCTR-IOR-17014170; http://www.chictr.org.cn).

The inclusion criteria were as follows: women aged 20–38 years, all embryos were vitrified with at least two high-quality day 3 embryos, and undergoing ET for the first time. The quality of cleavage-stage embryos was evaluated based on the number of blastomeres, blastomere symmetry, multinucleation, and fragmentation. A good quality day 3 embryo was defined as one with seven or eight blastomeres of equal size, no multinucleation, and <10% fragmentation, according to the Istanbul consensus (23). The exclusion criteria were FET cycles after pre-implantation genetic testing and patients with congenital or acquired uterine malformations, intrauterine adhesion, laparoscopic findings suggesting endometriosis, ultrasound findings suggesting adenomyosis, intramural uterine leiomyoma (≥3 cm), submucosal fibroids, scarred uterus, endometrial polyp, hydrosalpinx, PCOS, recurrent abortions (defined as three or more previous spontaneous pregnancy losses), abnormal results on parental karyotyping, and medical conditions that contraindicated assisted reproductive technology (ART) treatment or pregnancy.

### Randomization

On the second day of menstruation, eligible patients intending to undergo FET underwent blood testing for serum follicle-stimulating hormone (FSH), luteinizing hormone (LH), and estradiol (E2) and vaginal ultrasound evaluation. If there was no dominant follicle and E2 was <183 pmol/L, the patient was randomly allocated to group A (GnRHa-HRT group, *n* = 65) or group B (HRT group, *n* = 68) using computerized randomization codes prepared by an independent statistician, after the patient had provided informed consent. The randomized codes were enclosed in sequentially numbered, identical, opaque sealed envelopes that were opened sequentially after eligible patients were recruited and had signed the consent form. Neither patients nor investigators were blinded to patients’ group allocation because of the nature of the study.

### Treatment

Both centers used the same study protocol. Group A received a depot of long-acting GnRHa (3.75 mg triptorelin acetate; Ipsen Pharma Biotech, Paris, France), and 28 days later, patients underwent blood testing for serum FSH, LH, and E2 and an ultrasound scan to confirm complete pituitary desensitization. If there was no dominant follicle, and E2 was <183 pmol/L and FSH <5 IU/L, endometrial preparation was started with 6 mg (2 mg, three times daily) estradiol valerate (Delpharm Lille, Lys-lez-Lannoy, France) daily. Group B received 6 mg (2 mg, three times daily) estradiol valerate daily starting on day 2 of the menstrual cycle, with no previous pituitary suppression. In both groups, an ultrasound examination and blood test for serum progesterone were performed after 10–12 days. Exogenous estrogen supplementation was continued until 20 days if endometrial thickness was <8 mm. If after 20 days of oral estrogen supplementation the endometrial thickness was still <8 mm, ET was cancelled. After confirming endometrial thickness (≥8 mm), progesterone level (<1.2 ng/ml), and the absence of preovulatory follicles, progesterone 60 mg/day was administered by intramuscular injection on the same day.

On the fourth day of progesterone injection, two good quality day 3 frozen embryos were thawed and cultured for 2 h. Ultrasound-guided ET was performed by experienced clinicians at both centers. Luteal support was continued at least until a pregnancy test was performed 14 days after ET.

### Follow-Up and Data Collection

The implantation rate was calculated as the number of gestational sacs per number of transferred embryos. Clinical pregnancy was defined as the presence of an intrauterine gestational sac with a yolk sac, fetal pole, and fetal heart pulsations. After confirmation of clinical pregnancy, luteal support was continued until 10 weeks of gestation. Ectopic pregnancies were diagnosed by ultrasound or by laparoscopic visualization of an extrauterine gestational sac or the absence of an intrauterine gestational sac and increasing β-human chorionic gonadotropin levels following the failure of suction dilation and curettage to reveal products of conception. Live birth was defined as the birth of a live infant at ≥28 weeks of gestation.

### Sample Size Calculation

Sample size calculation was performed for the primary outcome, clinical pregnancy rate. In order to increase the rate from 50% to 60% with an alpha error of 5% and beta error of 90%, the sample size needed was 410 patients in both groups. At the time of registration, three reproductive centers planned to participate in the study, but for practical reasons, only two ultimately participated. We performed an early analysis after recruitment of nearly one-third of the patients. Based on the outcomes in the early analysis, we decided to discontinue further recruitment.

### Statistical Analysis

We performed an intention-to-treat (ITT) analysis in which patients were included in the group to which they had been randomly assigned regardless of the completeness of or adherence to the FET protocol. Differences between groups were evaluated with the two-tailed Student’s *t*-test if the data distribution passed the normality test; otherwise, the Mann–Whitney *U* test was used. The chi-squared test or Fisher’s exact test was used to compare the rates of fertilization, pregnancy, live birth, etc. *P*-values <0.05 were considered statistically significant.

## Results

### Characteristics of the Study Population

**Figure 1:**
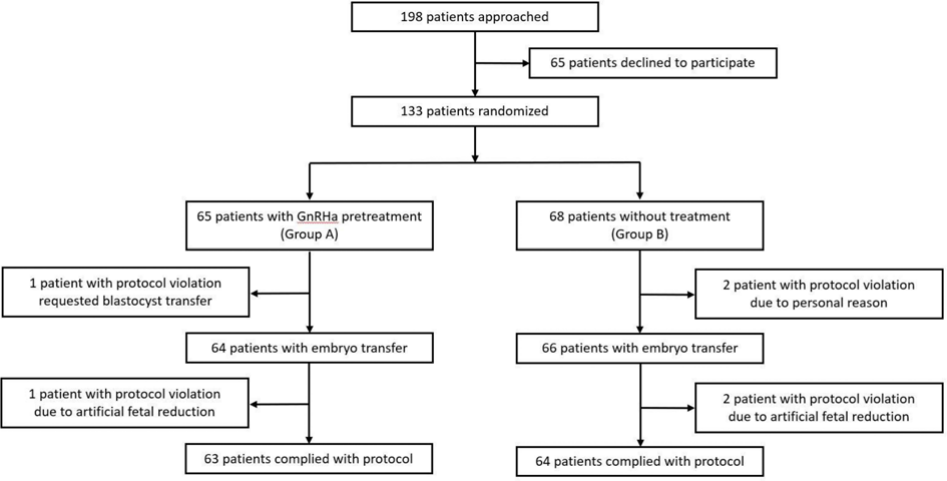
CONSORT diagram of participant screening, randomization, and follow-up.

A flow diagram of the participant selection is shown in **Figure 1**. A total of 198 patients were identified as eligible for participation in this trial. Of these patients, 133 provided written informed consent and were randomized. A total of 130 women (97.7%) underwent ET and 127 (95.4%) completed the protocols. The reasons for dropout are summarized in **Figure 1**.

The baseline characteristics of the included participants are summarized in **Table 1**. The age, body mass index (BMI), duration of infertility, infertility factors, anti-Müllerian hormone (AMH), antral follicular count, basic FSH level and days of estrogen supplementation prior to luteal phase induction, and the endometrial thickness were comparable between the two groups.

**Table 1:** Baseline characteristics (intention-to-treat analysis).

Item	Pretreatment (group A, *n* = 65)	Control (group B, *n* = 68)	*p*-value
Age (years)	30.6 ± 3.9	30.8 ± 4.3	0.8922
BMI (kg/m^2^)	21.2 ± 1.9	21.4 ± 2.7	0.6244
History of infertility (years)	3.6 ± 1.9	3.1 ± 2.6	0.2212
Primary infertility, *N* (%)	35 (53.8%)	35 (51.5%)	0.9198
Infertility factors, *N* (%)			0.7591
Tubal factors	37 (56.9%)	34 (50.0%)	
Male factors	25 (38.5%)	30 (44.1%)	
Recurrent IUI failure	3 (4.6%)	4 (5.9%)	
AMH (ng/ml)	5.4 ± 3.5	5.3 ± 2.6	0.4615
Antral follicular count	13.4 ± 3.5	14.3 ± 3.6	0.1468
bFSH	5.7 ± 1.2	6.0 ± 1.1	0.1348
Days of estrogen supplementation prior to luteal phase induction	11.4 ± 1.4	11.8 ± 1.3	0.1983
Endometrium thickness (mm)	9.5 ± 1.1	9.3 ± 1.2	0.1926

BMI, body mass index; IUI, intrauterine insemination; AMH, anti-Müllerian hormone; bFSH, basic follicle-stimulating hormone.

### FET Outcomes With HRT With or Without GnRHa

**Table 2** shows the clinical outcomes of FET. Based on the ITT analysis, groups A and B did not differ significantly in the clinical pregnancy rate (61.90% *vs*. 64.06%, *p* = 0.8012), implantation rate (47.62% *vs*. 45.31%, *p* = 0.7125), multiple pregnancy rate (51.20% *vs*. 41.46%, *p* = 3786), ectopic pregnancy rate (12.80% *vs*. 4.88%, *p* = 0.2088), and miscarriage rate (5.12% *vs*. 19.51%, *p* = 0.0910). There were also no significant differences between the two groups in the ongoing pregnancy rate (50.79% *vs*. 50.00%, *p* = 0.9287), live birth rate (49.21% *vs*. 50.00%, *p* = 0.9287), gestational week (37.8 *vs*. 38.3, *p* = 0.4218), and birth weight (2.63 *vs*. 2.80, *p* = 0.1478). There were no congenital anomalies in either group.

**Table 2:** Comparison of the pregnancy outcomes between groups (intention-to-treat analysis).

Item	Pretreatment (group A, *n* = 65)	Control (group B, *n* = 68)	*p*-value
Clinical pregnancy rate	39/65 (60.00%)	41/68 (60.29%)	0.8867
Implantation rate	60/126 (47.62%)	58/128 (45.31%)	0.7125
Ectopic pregnancy rate	5/39 (12.8%)	2/41 (4.88%)	0.2088
Miscarriage rate	2/39 (5.12%)	7/41 (19.51%)	0.0910
Multiple pregnancy rate	20/39 (51.20%)	17/41 (41.46%)	0.3786
Ongoing pregnancy rate	32/65 (50.79%)	32/68 (50.00%)	0.9386
Live birth rate	31/65 (49.21%)	32/68 (50.00%)	0.9199
Gestational week	37.8 ± 2.2	38.3 ± 2.5	0.4218
Birth weight (kg)	2.63 ± 0.55	2.80 ± 0.61	0.1478

## Discussion

Two key determinants of pregnancy outcomes following FET are embryo quality and endometrial receptivity. GnRHa-HRT is a modified HRT endometrium preparation method proven by patients suffering from endometriosis and adenomyosis (20, 21). However, it is not known whether GnRHa pretreatment is necessary with HRT-FET in patients with other causes of infertility.

Concrete conclusions regarding the potential benefit of HRT with GnRHa pretreatment cannot be drawn from previous RCTs. After analyzing the above research, we found that the inclusion and exclusion criteria for most RCTs were only regular menstruation or normal ovarian function, and the causes of infertility were also unclear (12, 13, 15–19). Additionally, embryo quality and number were not among the inclusion criteria in these RCTs (12–19).

To exclude embryologic influences on the pregnancy outcomes, in our study, we enrolled patients with at least two high-quality day 3 freeze-all embryos who were undergoing ET for the first time. The embryos were all vitrified and the survival rate was 100% after thawing. In contrast, in previous RCTs, the embryos were frozen slowly (12–15); the possibility that damage to the embryos during this process may have affected the pregnancy outcomes cannot be ruled out. Additionally, we excluded factors that could have a negative impact on pregnancy outcomes such as congenital or acquired uterine malformations, intrauterine adhesion, laparoscopic findings suggesting endometriosis, ultrasound findings suggesting adenomyosis, intramural uterine leiomyoma (≥3 cm), submucosal fibroids, scarred uterus, endometrial polyp, and hydrosalpinx. These stringent criteria made the two groups more comparable.

We compared the pregnancy outcomes of women who underwent HRT-FET with or without GnRHa pretreatment. These women were undergoing IVF treatment mainly because of tubal or male factors or unexplained infertility. In line with most published RCTs, we found that pretreatment with GnRHa did not improve the clinical pregnancy rate, implantation rate, live birth rate, early pregnancy loss rate, ectopic pregnancy rate, or birth outcomes, which were comparable between the two protocols. These results are consistent with those of a recent Cochrane meta-analysis reporting similar clinical pregnancy and miscarriage rates after FET between women with *vs*. without GnRHa pretreatment (24).

There are a few possible reasons why there was no benefit from GnRHa pretreatment in our RCT. Firstly, the results of a previous study suggest that GnRHa pretreatment improved the reproductive outcomes by suppressing recessive ovulation (12). However, in the present study, both groups received 6 mg estradiol valerate daily starting from day 2 of the menstrual cycle. None of the patients had cycle cancellation due to follicular development, and the hormone levels before endometrium transformation were normal. Secondly, most mechanistic studies that have demonstrated improved endometrium receptivity were carried out in animals (7, 25). Thirdly, GnRHa pretreatment was shown to improve the reproductive outcomes of patients with endometriosis (20, 21). We speculated that some patients with mild, undiagnosed endometriosis may benefit from GnRHa pretreatment. However, a recent meta-analysis showed that downregulation was effective only for patients with stage III or IV endometriosis, but not for those with mild endometriosis (26).

There are also some disadvantages to the GnRHa pretreatment protocol, such as the high cost (22), risk of hypo-estrogenic side effects before hormone replacement (27), ovarian cyst formation (13, 22, 28), and the time-consuming preparation process.

There were some limitations to the present RCT. Firstly, we performed an early analysis after recruitment of nearly one-third of patients. Because two high-quality cleavage-stage embryos were transferred, the twin pregnancy rate was as high as 46.3% and there was no significant difference in the pregnancy rates between the two groups. Recently, blastocyst culture and single blastocyst transfer have markedly decreased the multiple pregnancy rate without significantly reducing the clinical pregnancy rate. Based on the outcomes of the early analysis and for patient safety, we decided to discontinue further recruitment. Another potential shortcoming of the current trial was the lack of blinding and the absence of a control arm treated with placebo. Finally, pregnancy-related complications and neonatal outcomes were not analyzed as these data were collected by telephone follow-up and could not be verified.

## Conclusion

The results of this study demonstrate that pretreatment with GnRHa does not improve the reproductive outcomes for women receiving HRT-FET.

## Data Availability Statement

The raw data supporting the conclusions of this article will be made available by the authors, without undue reservation.

## Ethics Statement

The studies involving human participants were reviewed and approved by the Ethics Committee of Guangzhou Women and Children’s Hospital. The patients/participants provided written informed consent to participate in this study. Written informed consent was obtained from the individual(s) for the publication of any potentially identifiable images or data included in this article.

## Author Contributions

LS and PL were responsible for the conception and design of the study and the interpretation of data, revised the article critically for important intellectual content, and approved the final draft for publication. JX and S-ZL contributed to the collection, analysis, and interpretation of data and drafted and revised the whole article. M-NY and P-LL contributed to data collection and drafted and revised the article for important intellectual content. All authors contributed to the article and approved the submitted version.

## Conflict of Interest

The authors declare that the research was conducted in the absence of any commercial or financial relationships that could be construed as a potential conflict of interest.

## Publisher’s Note

All claims expressed in this article are solely those of the authors and do not necessarily represent those of their affiliated organizations, or those of the publisher, the editors and the reviewers. Any product that may be evaluated in this article, or claim that may be made by its manufacturer, is not guaranteed or endorsed by the publisher.
